# A Validation Tool (VaPCE) for Postcoordinated SNOMED CT Expressions: Development and Usability Study

**DOI:** 10.2196/67984

**Published:** 2025-02-28

**Authors:** Tessa Ohlsen, Viola Hofer, Josef Ingenerf

**Affiliations:** 1 Section for Clinical Research IT, Institute of Medical Biometry and Statistics, University of Luebeck and University Hospital Schleswig-Holstein, Luebeck, Germany; 2 Institute of Medical Informatics, University of Luebeck, Lübeck, Germany; 3 Thieme Compliance GmbH, Erlangen, Germany

**Keywords:** SNOMED CT, PCE, postcoordination, FHIR, validation, postcoordinated expression, Fast Healthcare Interoperability Resource

## Abstract

**Background:**

The digitalization of health care has increased the demand for efficient data exchange, emphasizing semantic interoperability. SNOMED Clinical Terms (SNOMED CT), a comprehensive terminology with over 360,000 medical concepts, supports this need. However, it cannot cover all medical scenarios, particularly in complex cases. To address this, SNOMED CT allows postcoordination, where users combine precoordinated concepts with new expressions. Despite SNOMED CT’s potential, the creation and validation of postcoordinated expressions (PCEs) remain challenging due to complex syntactic and semantic rules.

**Objective:**

This work aims to develop a tool that validates postcoordinated SNOMED CT expressions, focusing on providing users with detailed, automated correction instructions for syntactic and semantic errors. The goal is not just validation, but also offering user-friendly, actionable suggestions for improving PCEs.

**Methods:**

A tool was created using the Fast Healthcare Interoperability Resource (FHIR) service $validate-code and the terminology server Ontoserver to check the correctness of PCEs. When errors are detected, the tool processes the SNOMED CT Concept Model in JSON format and applies predefined error categories. For each error type, specific correction suggestions are generated and displayed to users. The key added value of the tool is in generating specific correction suggestions for each identified error, which are displayed to the users. The tool was integrated into a web application, where users can validate individual PCEs or bulk-upload files. The tool was tested with real existing PCEs, which were used as input and validated. In the event of errors, appropriate error messages were generated as output.

**Results:**

In the validation of 136 PCEs from 304 FHIR Questionnaires, 18 (13.2%) PCEs were invalid, with the most common errors being invalid attribute values. Additionally, 868 OncoTree codes were evaluated, resulting in 161 (20.9%) PCEs containing inactive concepts, which were successfully replaced with valid alternatives. A user survey reflects a favorable evaluation of the tool’s functionality. Participants found the error categorization and correction suggestions to be precise, offering clear guidance for addressing issues. However, there is potential for enhancement, particularly regarding the level of detail in the error messages.

**Conclusions:**

The validation tool significantly improves the accuracy of postcoordinated SNOMED CT expressions by not only identifying errors but also offering detailed correction instructions. This approach supports health care professionals in ensuring that their PCEs are syntactically and semantically valid, enhancing data quality and interoperability across systems.

## Introduction

### Background

The ongoing digitalization in medicine has significantly increased the amount of available medical data in the health care sector. To optimize medical care, it is crucial to use this data efficiently. For this, it is essential that the data can be exchanged and processed automatically across different systems. This requires not only technical compatibility but also semantic interoperability. Semantic interoperability ensures that the meaning of the data is maintained when it is transferred to another system. A key aspect of semantic interoperability is the sophisticated use of suitable coding systems and medical standards for medical terms [1]. One example is the terminology SNOMED Clinical Terms (SNOMED CT). With over 360,000 concepts, SNOMED CT is considered the most comprehensive terminology in medicine. SNOMED CT facilitates machine-readable data exchange and reduces issues related to country-specific and domain-specific coding, as well as difficult versioning issues. However, despite its comprehensiveness, SNOMED CT cannot cover every possible medical circumstance by precoordinated concepts alone due to the complexity of natural language [2]. For instance, an allergy to metals: The SNOMED CT International Edition, version 2024-09-01, lists a total of 1575 different metals. However, only 43 specific allergies to metals are included in this edition and version of SNOMED CT. This indicates that not every allergy-relevant metal or substance can be expressed through a precoordinated concept, such as an allergy to Cobalt-chromium alloy. To address such gaps, SNOMED CT offers postcoordination in contrast to many other vocabularies. Here, existing precoordinated concepts are combined into new expressions using formal grammar. This avoids the well-known problem of combinatorial explosion [2]. An example postcoordinated expression (PCE) for an allergy to a Cobalt-chromium alloy based on Compositional Grammar [3] is as follows:

1155942004 |Allergy to metal and/or metal compound|:

{719722006 |Has realization| = 472964009 |Allergic process|,

246075003 |Causative agent| = 256526003 |Cobalt-chromium alloy|}.

Here, the focus concept Allergy to metal and/or metal compound is refined by combining attributes (eg, Causative agent) with corresponding attribute values (eg, Cobalt-chromium alloy) as attribute relations.

Creating PCEs improves the detailed recording of medical facts and enhances patient record quality and automated processing. PCEs are complex due to the syntactic requirements of the Compositional Grammar [3] and the semantic rules of the Concept Model [4]. The syntactic requirements ensure that PCEs are correctly structured, while the semantic rules ensure that they accurately represent medical concepts and their relationships. This complexity makes it challenging to create valid accurate PCEs [2].

**Figure 1.**
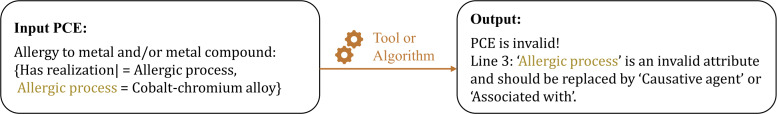
For every invalid PCE, a correction suggestion should be made automatically using the developed tool. PCE: postcoordinated expression.

Since Germany acquired a license for SNOMED CT in January 2021, the interest in using SNOMED CT has grown. SNOMED CT is increasingly being used in various national projects [5,6]. Despite this, postcoordinated SNOMED CT expressions are still used hesitantly. Nevertheless, PCEs are used in the Medical Information Objects of the National Association of Statutory Health Insurance Physicians [7] or in anamnesis forms from Thieme Compliance [8]. Nonetheless, some do not fully comply with the rules of the Compositional Grammar or the Concept Model. To address these issues, this work aims to develop a tool for the validation of PCEs. The focus is not only on determining whether a PCE is correct but also on providing detailed information and concrete suggestions for correcting a PCE (Figure 1). These suggestions are based on the analyzed errors and provide targeted recommendations for correcting specific PCEs.

### Related Work

Currently, SNOMED International provides a tool called “SNOMED International APG Parser: SNOMED CT Compositional Grammar” which validates PCEs based on syntactic rules of Compositional Grammar [9]. Nevertheless, this tool only assesses syntactic correctness and does not consider the semantic rules of the Concept Model, which are essential for ensuring contextually accurate PCEs. This work aims to supplement this by adding semantic validation, which should provide better feedback for the user.

An alternative method for the syntactic and semantic validation of PCEs is using the commercial terminology server Ontoserver [10] of Commonwealth Scientific and Industrial Research Organization. Ontoserver is currently the only Fast Healthcare Interoperability Resource (FHIR) terminology server that supports postcoordination at all. Ontoserver is used to validate the PCEs in combination with the FHIR service $validate-code [11]. This checks a PCE against specific coding systems, such as SNOMED CT. This method provides a validation result through a Representational State Transfer (REST) request that returns a JSON object [11]. Although this service indicates errors, it presents them in a technical manner that requires a solid understanding of SNOMED CT. This work’s tool will build upon the FHIR service $validate-code based on Ontoserver to offer more comprehensive validation and suggestions. Hence, the difference between this work and the Ontoserver is that the focus is on the individual correction instructions for each PCE.

## Methods

### Overview

**Figure 2.**
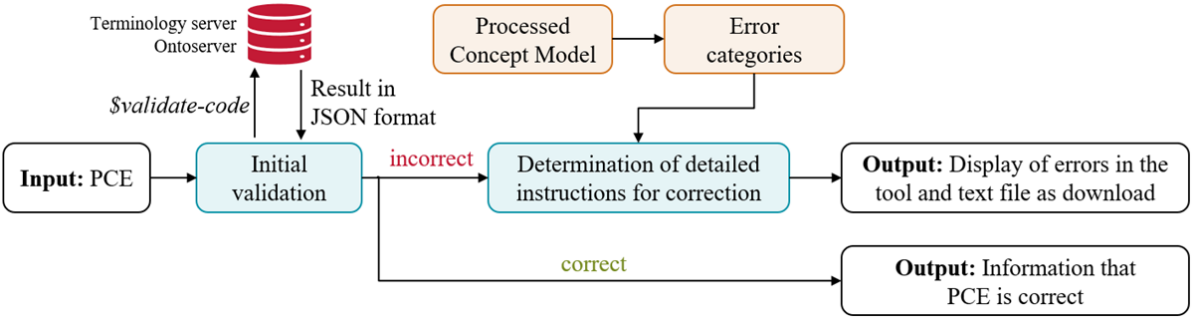
Pipeline for the validation of a PCE. PCE: postcoordinated expression.

This work aims to develop a user-friendly tool for validating postcoordinated SNOMED CT expressions. The focus is on the automated provision of detailed correction instructions if a PCE contains syntactic or semantic errors. The tool’s pipeline is illustrated in Figure 2. First, a PCE is validated using the FHIR service $validate-code [11] and the terminology server Ontoserver [10]. If the entered PCE is correct, this is communicated to the user, and the algorithm terminates. For incorrect PCEs, the tool generates detailed correction instructions in the next step, based on the results of the FHIR service. A processed Concept Model in JSON format and custom error categories are also used for this purpose. The correction instructions are presented to the user. The individual components of the pipeline are explained in more detail below.

### Initial Validation

**Figure 3.**
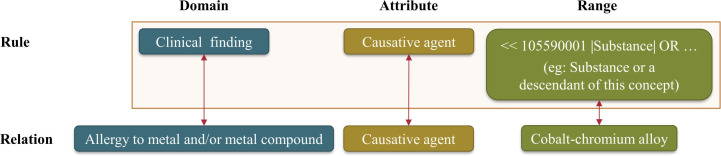
Domain and range example for the SNOMED CT attribute Causative agent.

FHIR, an interoperable data standard developed by Health Level 7 [1], is gaining increasing importance both nationally [12] and internationally [13,14]. In addition to the exchange of patient-related health information, FHIR also offers a terminology module with various services [1]. In this project, the FHIR service $validate-code [11] combined with Ontoserver [10] plays a central role. This is used to verify the syntactic and semantic correctness of a postcoordinated SNOMED CT expression, in combination with a terminology server. The $validate-code service operates via an HTTP POST request. The response is a JSON object that contains the results of the validation. Figure 3 shows an excerpt from this JSON object for an invalid PCE. The error messages indicate that the concept 472964009 |Allergic process| is not a valid attribute. This detailed output is attributed to the commercial Ontoserver, which possesses extensive knowledge of SNOMED CT, enabling precise analysis of PCEs.

Although the output is focused on automatic processing rather than human readability, it remains particularly valuable due to this specialization. This output provides a crucial basis for the next stages of the tool’s pipeline.

### Determination of Detailed Instructions for Correction

#### Processed Concept Model

**Figure 4.**
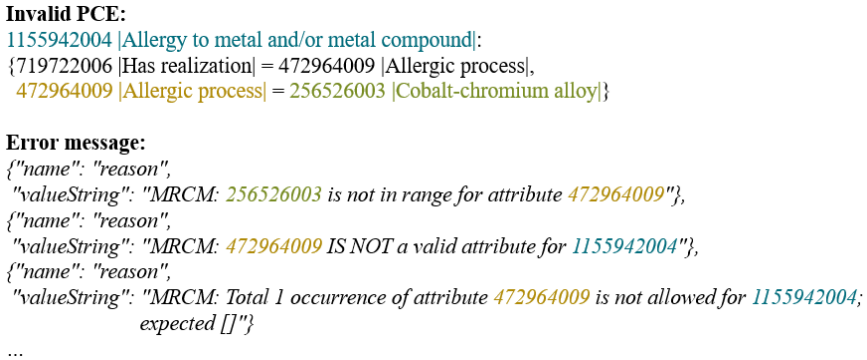
Extract of an error message for an invalid PCE based on an Ontoserver request. PCE: postcoordinated expression.

The Concept Model defines rules for how SNOMED CT concepts and PCEs are structured using formal logic and specific guidelines. These rules ensure that medical expressions are meaningful and consistent. For each SNOMED CT attribute, the Concept Model specifies a domain and a range (Figure 4):

- Domain: This includes concepts (eg, Allergy to metal and/or metal compound) from at least one of the 19 Top-Level Hierarchies (eg, Clinical finding).
- Range: This specifies a subset of SNOMED CT concepts that are valid values for an attribute. For the attribute Causative agent, for instance, only certain substances, such as Cobalt-chromium alloy, are included in the range.

Additionally, the Concept Model specifies the cardinality of the attributes and whether attributes must be grouped [2,4].

**Figure 5.**
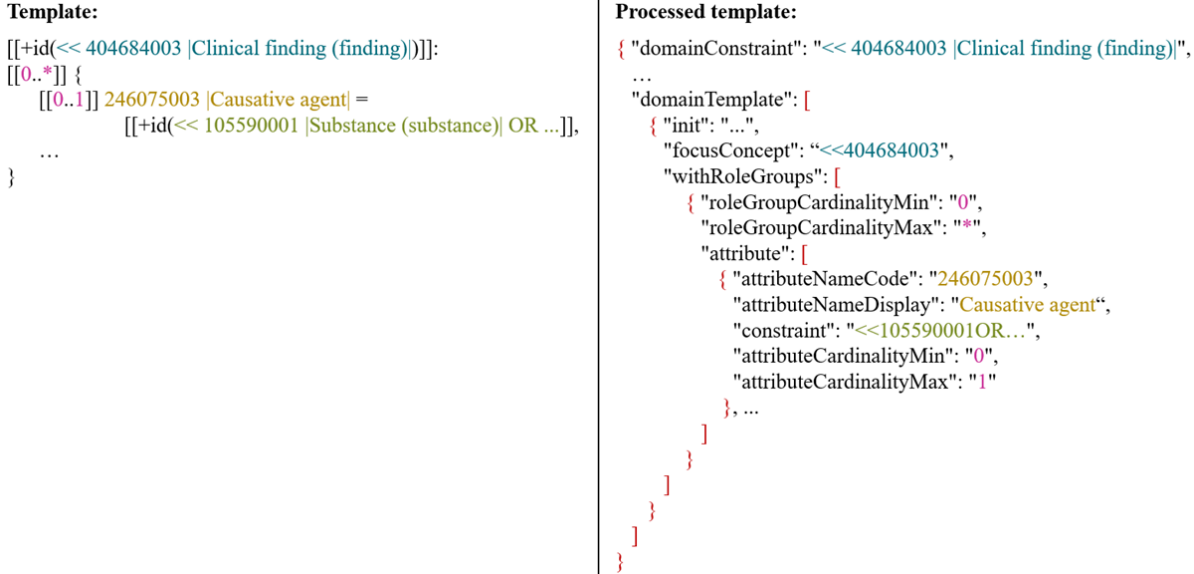
Extract from the processed machine-readable Concept Model in JSON format which proves to be advantageous for further processing.

To facilitate the tool, a machine-readable Concept Model (MRCM) is included in the RF2 files for each SNOMED CT edition and version [15]. For our purposes, the MRCM Domain Reference Set (International Edition, 2024-05-01) is used. The MRCM Domain Reference Set is provided as a text file, with each domain represented by a detailed entry. Each entry includes all essential information about the respective domain, including a so-called template that is particularly significant for this work [15,16]. An excerpt from one of these templates is shown on the left side of Figure 5. The syntax of the templates is based on Compositional Grammar and includes the relevant attributes, as well as the cardinalities and value ranges for each domain. To further enhance usability, we developed an algorithm that decomposes the template into its individual components and presents this information in JSON format. This allows for a structured and efficient processing of the template components in the next step. All relevant information is already available in the appropriate format, enabling subsequent processes to use it directly without any further conversions. An extract of the JSON is displayed on the right side of Figure 5. The JSON is available on GitHub [17].

#### Error Categories

##### Overview

**Figure 6.**
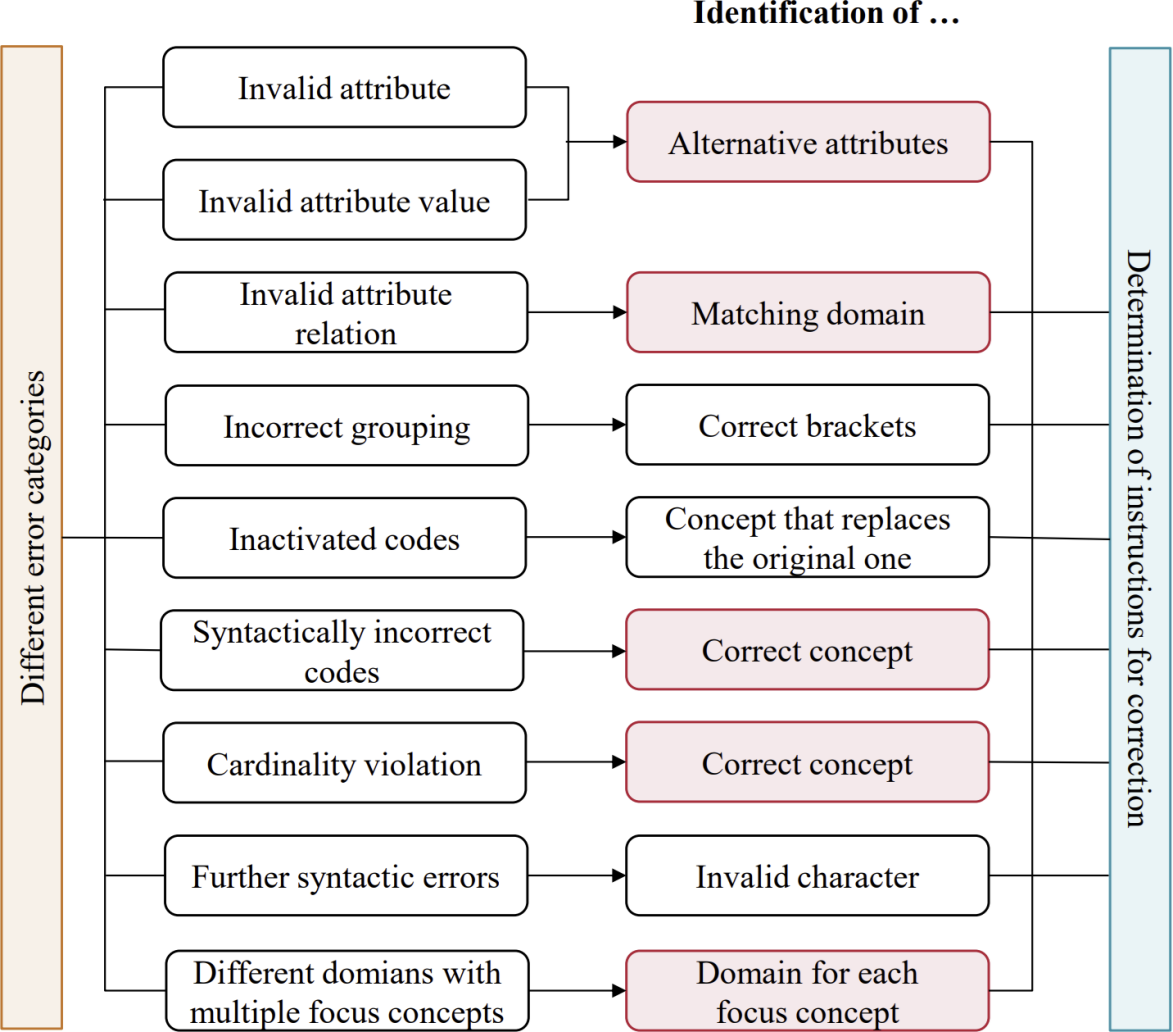
An overview of the various error categories (left side) and the corresponding correction focus (right side). For all categories, the processed Concept Model in JSON format plays a certain role. For the correction instructions highlighted in red, a terminology server and FHIR services are also used.

Based on the authors’ expertise in SNOMED CT postcoordination [2,18-20], a total of nine error categories have been identified. For each error category, specific guidelines have been developed and are provided for an individual PCE. A general overview of the error categories and the focus of the guidelines is shown in Figure 6. The following sections provide a detailed description of each error category. Examples of the individual error categories are available on GitHub [21].

##### Invalid Attribute

If a SNOMED CT concept is used that does not belong to the valid 126 attributes or is not valid in a specific domain, it is categorized under this category.

###### Correction Instruction

The solution involves identifying a valid SNOMED CT attribute. Users are provided with information about the domain of the focus concept, along with definitions and value ranges for alternative attributes. This helps to replace the invalid attribute with a suitable one. If no appropriate alternative is found, or if no attributes exist within the domain, the user must either modify the attribute value or create a new PCE with a different focus concept. For the invalid PCE shown in Figure 2, this error category is identified. The algorithm would also determine that the incorrect attribute Allergic process should be replaced with Causative agent or Associated with.

###### Technical Implementation

To implement this, the FHIR service $expand is used in combination with a terminology server and the Expression Constraint Language (ECL) of SNOMED CT [22]. The ECL in this case defines the value range of an attribute, such as << 105590001 |Substance| OR .... This allows for verifying whether the current attribute value is included in the provided list. If an alternative attribute is identified, the PCE is revalidated to check for any additional errors.

### Invalid Attribute Value

In this case, the chosen attribute value is not in the value range for the attribute.

#### Correction Instruction

For manually created PCEs, the attribute value is typically chosen correctly as it directly relates to a specific medical circumstance. However, the chosen value might not be within the valid range. The focus is on identifying appropriate alternative attribute values. Users will receive similar guidance as in the “Invalid Attribute” error category, including details on the acceptable value range and potential alternatives.

#### Technical Implementation

The process adheres to the same principle outlined in the “Invalid Attribute” section.

### Invalid Domain

In this error category, the attribute relation (comprising an attribute and its value) is semantically correct but does not match the domain of the focus concept.

#### Correction Instruction

Achieving a semantically correct relation requires a different domain of the focus concept. Users are provided with a list of domains where the current attribute relation is valid, enabling them to modify the focus concept accordingly.

#### Technical Implementation

The appropriate domain is determined using the FHIR service $expand and a terminology server. The request is based on an ECL that specifies the domain constraint from the Concept Model. For instance, an expression might be *<<* 404684003 |Clinical finding*|*. If the focus concept appears in the results returned by this query, it belongs to the corresponding domain.

### Different Domains With Multiple Focus Concepts

#### Overview

According to the Compositional Grammar, it is valid to use multiple focus concepts connected by “+” (AND). If multiple focus concepts from different domains are used—such as Clinical finding and Procedure—this results in a semantic error. In such cases, the PCE is categorized under this error category.

#### Correction Instruction

The domains of the focus concepts used are displayed.

#### Technical Implementation

We determined the Concept Model Domain performed as described in the “Incorrect attribute relation” category.

### Incorrect Grouping

#### Overview

This category includes errors related to the bracketing of RoleGroups, which are crucial for the syntactic and semantic integrity of PCEs. Common issues involve missing or incorrect brackets, as well as imbalances between open and closed brackets.

#### Correction Instruction

Users are provided with detailed information on which attributes are incorrectly grouped and where bracket corrections are necessary. Specific guidance is given on how to fix incorrectly placed brackets.

#### Technical Implementation

A straightforward analysis of the error message and the PCE is conducted to provide targeted correction instructions. The error message shows the position and the concept before which the error occurs. For example, it may indicate that the character “{” is missing, suggesting that a RoleGroup is missing and should be started at the specified location. In this case, the tool would also determine the position where the RoleGroup should be closed again. Technically, this is done through simple processing of the strings for the error message, as well as the JSON object of the PCE.

### Inactivated Codes

This error category pertains to PCEs that include codes that are no longer valid or active in the current version and have been replaced by others.

#### Correction Instruction

Users are provided with an alternative concept that is semantically equivalent to the inactivated concept.

#### Technical Implementation

Identifying active concepts for inactivated codes is part of version management. SNOMED International offers Historical Association Reference Sets (RefSets) for this purpose. In this work, the following two RefSets are used:

- 900000000000527005 | SAME AS association reference set|: For identifying semantically equivalent active concepts.
- 900000000000526001 | REPLACED BY association reference set|: For linking deactivated concepts that have been replaced by new concepts.

These RefSets can be integrated into ECL. Additionally, ECL provides member filters that can be used in combination with respective RefSets, for example:

^ [targetComponentId] 900000000000526001 | REPLACED BY association reference set|

{{ M referencedComponentId = 224799004 |Unfamiliar environment (environment)| }}.

This ECL queries the REPLACED BY association reference set to identify the concept that has replaced the inactivated concept 224799004 |Unfamiliar environment (environment)|. In this case, the result would be 1186687002 |Unfamiliar environment (finding)|.

### Syntactically Incorrect Codes

#### Overview

This error category refers to SNOMED CT Identifiers that cannot be linked to any valid SNOMED CT concept. The issue is not that the concept is inactive, but rather that the identifier is missing characters.

#### Correction Instruction

Identifying a valid SNOMED CT concept that corresponds to the numerical sequence of the incorrect identifier is necessary for correcting a PCE. The user will then be provided with this concept.

#### Technical Implementation

For this category, the FHIR service $expand, a terminology server, and ECL are also used. The ECL expression *<<* 138875005 |SNOMED CT Concept| is used to return all SNOMED CT concepts. Subsequently, all concepts containing the specified numeric sequence are identified. These concepts are then validated using the FHIR service $validate-code to determine which one fits both syntactically and semantically with the original identifier.

### Cardinality Violation

#### Overview

As previously mentioned, the Concept Model defines cardinalities that specify the minimum and maximum occurrences of an attribute. A PCE is categorized in this category if it does not adhere to these cardinality constraints.

#### Correction Instruction

Initially, the user is shown the attribute that violates the cardinality constraints, along with the cardinalities defined for this attribute in the Concept Model. If there is a violation of the minimum cardinality, the attribute must be added. In case of a violation of the maximum cardinality, either a grouped attribute should be moved to an additional RoleGroup or ungrouped attributes should be deleted.

#### Technical Implementation

A basic analysis of the error message and the PCE is performed using the processed Concept Model in JSON format. The error message precisely indicates which attribute violates a cardinality (minimum or maximum) constraint. This information is automatically extracted from the JSON. Using the JSON object, an appropriate error correction can be easily determined.

### Further Syntactic Errors

#### Overview

If a PCE contains characters that do not conform to the Compositional Grammar rules, it falls into this category. For instance, using the symbol “:@” instead of “:” before refinement would trigger an error indicating that the symbol “@” is not allowed.

#### Correction Instruction

The instructions specify which character needs to be replaced and the exact position where the replacement should occur.

#### Technical Implementation

A fundamental analysis of the error message and the PCE is conducted to provide accurate correction instructions. Through string processing and parsing, the position and the incorrect character are also extracted here.

## Results

### Validation Tool

The validation tool has been seamlessly integrated into the existing web application WASP [18], which was developed in a previous project. Accessible to the public via [23], the application enables users to use the new tool through the “Validation” option in the main menu. Users can choose to validate a single PCE directly or upload a file for validation. The tool supports various formats, including FHIR Questionnaires [24] and FHIR ValueSets [25] in JSON format, as well as CSV files, offering a flexible and versatile approach for validating different types of data.

**Figure 7.**
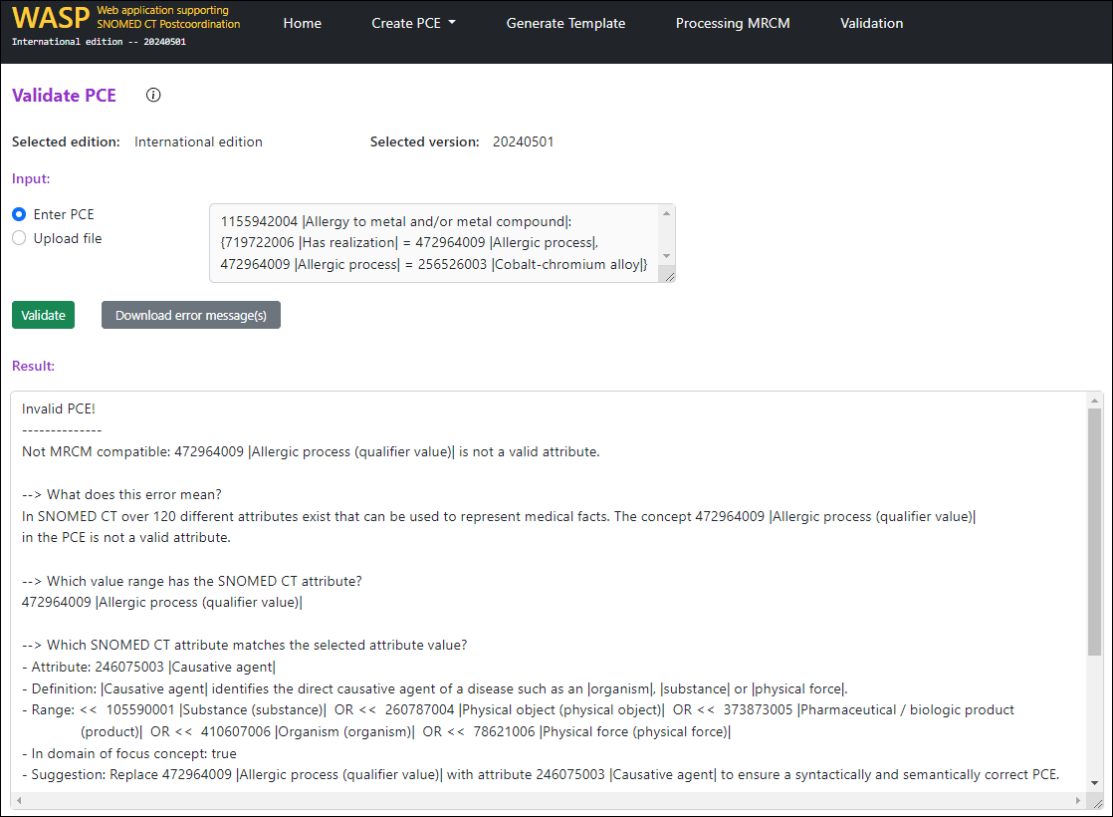
Extract from the tool for validating the postcoordinated SNOMED CT expressions.

Upon completing the validation, users receive a comprehensive overview of any errors detected during the process. These errors are clearly displayed on the user interface, ensuring easy traceability and resolution. Additionally, users have the option to download the error reports as a text file, enabling further analysis or optimization when needed. A screenshot of the web application, highlighting the user interface and layout, is shown in Figure 7.

### Use Case 1: Existing PCEs in FHIR Questionnaires

For the validation of the tool, real existing postcoordinated SNOMED CT expressions were used to establish a practical and reliable testing environment. These PCEs were incorporated into the FHIR Questionnaires developed by Thieme Compliance, which are designed for modeling anamnesis forms. Thieme Compliance offers these anamnesis forms in multiple languages to enhance usability for both patients and medical professionals. For machine processing and analysis, on the other hand, language-independent codes are used, ensuring precise and efficient data evaluation regardless of the language used [8]. This validation specifically focused on the German-language anamnesis forms as FHIR Questionnaires [24].

In total, 1122 postcoordinated SNOMED CT expressions were identified across 303 FHIR Questionnaires. Since many of these PCEs were repeated, 136 unique PCEs were distinguished. The validation results using the developed tool were as follows:

- 118 (86.8%) correct PCEs
- 18 (13.2%) invalid PCEs

In addition to identifying the correct and invalid PCEs, the developed algorithm carried out a detailed error categorization, uncovering six distinct error categories as shown in Table 1. These errors were analyzed in collaboration with the coders from Thieme Compliance. It was confirmed that all errors were correctly identified, and the correction suggestions were content-wise accurate.

**Table 1.** Various error categories and their frequency in the 18 invalid PCEs^a^.

Error categories	Frequency
Invalid attribute value	9
Invalid grouping	2
Inactivated codes (International Edition, 2024-05-01)	3
Syntactically incorrect codes	1
Cardinality violation	2
Further syntactic error	1

a PCE: postcoordinated expression.

### Use Case 2: Existing PCEs in FHIR ConceptMaps

In a previous project, ICD-O tuples were mapped to the OncoTree, using SNOMED CT as an intermediary step [20]. All 868 OncoTree codes were represented as postcoordinated SNOMED CT expressions based on the International Edition, 2021-01-31 in a FHIR ConceptMap [26]. The developed tool is now being used to validate all PCEs. Since the ConceptMap is from 2021, it was expected to contain inactive concepts. If this is the case, they will be replaced by active concepts from the International Edition, dated May 1, 2024 [20]. Any other errors will also be corrected accordingly.

Using the tool, 161 (20.9%) out of 868 PCEs were identified, each containing a concept that is no longer present in the International Edition, 2024-01-05. Some of these inactive concepts occurred multiple times, leading to the identification of 79 distinct inactive concepts. For all inactive concepts, an equivalent active SNOMED CT concept was found and was manually validated by an SNOMED CT expert, ensuring that the PCEs in the newer version are once again both semantically and syntactically correct. In addition, no other causes of error could be identified in the data.

### Usability Survey

For the user survey, the participants received four real existing invalid PCEs along with the corresponding error messages. It was ensured that each was assigned to a different error category (Multimedia Appendix 1). The following error categories were used:

- Syntactically incorrect code
- Inactivated code
- Invalid attribute
- Invalid attribute value

To select these categories, PCEs from the literature were reviewed and analyzed. Additionally, discussions were held with professionals actively involved in coding PCEs. This allows us to identify the error categories most commonly causing issues in practice.

In addition, the authors developed a customized questionnaire with ten questions that assessed both the self-evaluation of SNOMED CT knowledge and the overall user experience with the error messages. For nine of the questions, an ordinal scale with five levels ranging from 1=worst to 5=best rating was used, while the last question was open-ended. None of the questions were mandatory, and no answer options were preselected. The complete questionnaire with the answer options is displayed in Multimedia Appendix 1. The survey was conducted with five employees of Thieme Compliance, all of whom have basic knowledge of SNOMED CT postcoordination. Each participant was given the four invalid PCEs along with the corresponding error messages. Subsequently, the participants completed the questionnaire anonymously.

**Figure 8.**
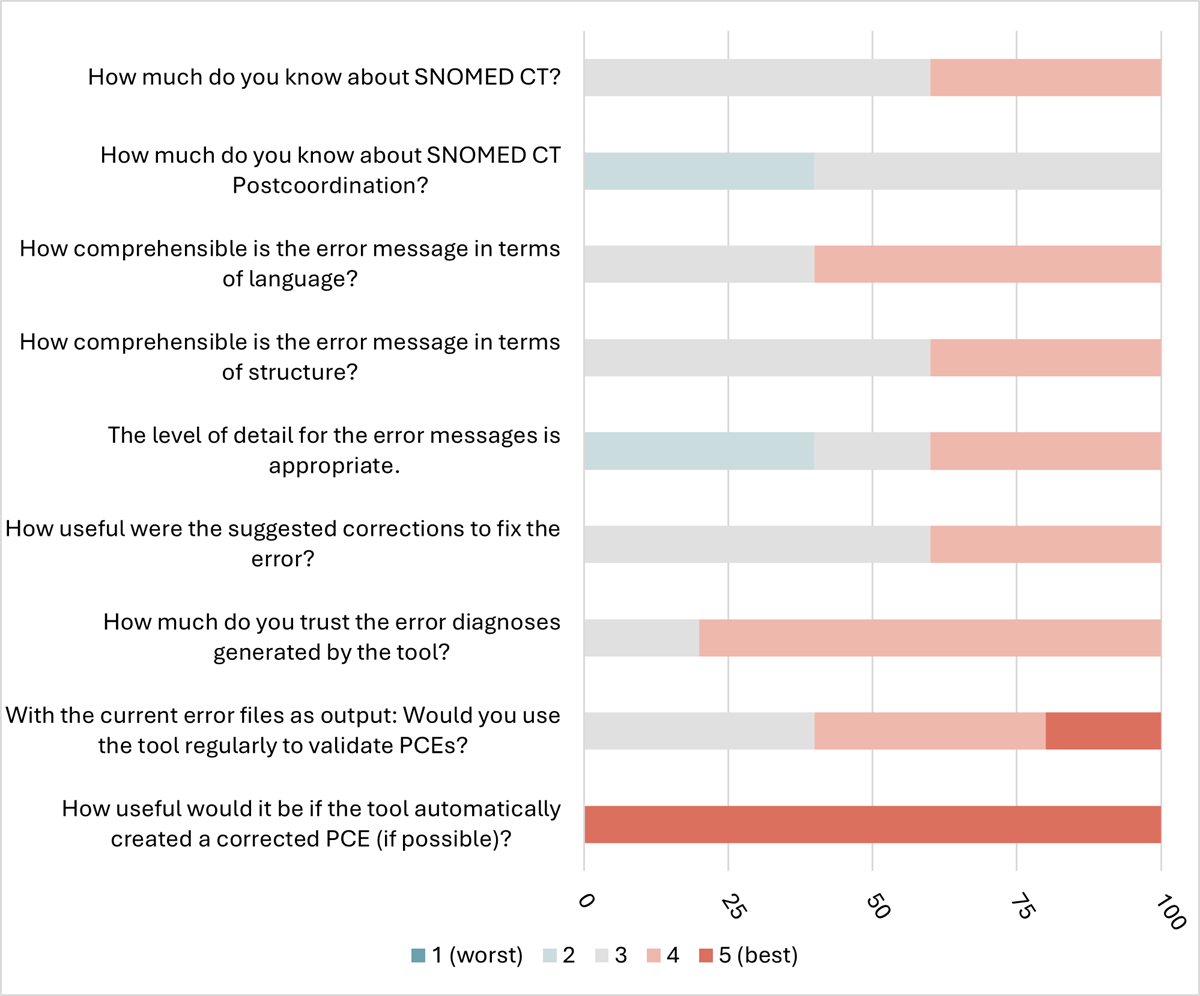
Results of the usability survey. PCE: postcoordinated expression.

The chart in Figure 8 summarizes the survey results on various aspects of the tool’s functionality. Overall, the tool received predominantly positive feedback, as indicated by the fact that only one question garnered slightly negative ratings from two participants. The comprehensibility of the error messages—both in terms of language and structure—was rated with high or neutral scores, with the structure being assessed somewhat more critically than the language. Opinions were divided regarding the level of detail in the error messages: two participants found the level appropriate, another participant gave a neutral rating, and two participants provided less favorable feedback. These were also documented in the free-text comments. Despite these criticisms, trust in the tool and the readiness to use it regularly for PCE validation remain high, as reflected in the overall positive ratings. Additionally, all participants rated the tool’s potential expansion to include automatic PCE correction functionality with the highest score of 5. In summary, the chart demonstrates a positive assessment of the tool’s functionality, though there is room for improvement in areas such as the level of detail in the error messages.

## Discussion

### Principal Findings

In this work, a tool for validating postcoordinated SNOMED CT expressions was developed that detects both syntactic and semantic errors. Incorrect PCEs are classified into one of the nine developed error categories, enabling the user to receive targeted feedback. The key added value and focus of the tool lies in generating specific correction suggestions for each identified error.

The successful integration of this tool into the existing web application WASP [23] was confirmed through tests with real existing PCEs in FHIR Questionnaires [24] and an FHIR ConceptMap [26]. The use of these PCEs, provided in the FHIR Questionnaires from Thieme Compliance [8], demonstrated that the tool can reliably verify the correctness and validity of expressions. This is particularly relevant for accurate modeling of medical data and its integration into electronic health records. Additionally, the error files of the 18 PCEs were provided to Thieme Compliance for correction. Another use case, the validation of PCEs in the context of a mapping between ICD-O and OncoTree [20], highlights the tool’s flexibility and usefulness in identifying and replacing inactive concepts. The ability to replace outdated concepts with current ones is crucial for the quality and relevance of medical data. The tool contributes to enabling cross-version analyses. It offers a solution for legacy data coded with earlier versions of SNOMED CT, where certain concepts may no longer exist. In such cases, the developed tool can assist in replacing these concepts with current equivalents.

A user survey has indicated that there is both interest and trust in the application. The results show a generally positive evaluation of the tool’s functionality, although there is some potential for improvement regarding the level of detail in the error messages. In fact, 50% of participants rated the messages as too complex. Considering this, the development of the tool will place a special emphasis on revision. The plan is to allow users to choose between a newly developed, concise error message, and a more detailed version.

A comparison with existing methods, such as the SNOMED International APG Parser [9] and the FHIR service $validate-code [11] combined with the terminology server Ontoserver [10], shows that the developed tool adopts a more comprehensive validation approach, placing particular emphasis on providing correction suggestions. While the APG Parser primarily checks syntactic correctness and the $validate-code service is limited to technical errors, the developed tool goes a step further: it enables in-depth error analysis, allowing for more precise corrections. This ensures that PCEs are created not only with correct syntax but also with accurate semantics. As a result, the quality and applicability of these expressions in practice are significantly improved.

A key aspect of this work is that the tool should not be understood as an automatic system for improving PCEs; rather, it provides targeted suggestions for the manual optimization of expressions. As the results of the user survey indicate, this would be a worthwhile feature. Automated corrections are often difficult to implement, as there is rarely a single correct solution. For instance, the tool suggests alternative attributes for the example PCE of Figure 1:

1155942004 |Allergy to metal and/or metal compound|:

{719722006 |Has realization| = 472964009 |Allergic process|,

472964009 |Allergic process| = 256526003 |Cobalt-chromium alloy|} such as:

- Causative agent
- Associated with

In this case, the attribute Causative agent should be used because this attribute is typically used in combination with allergies. A system that autonomously selects the appropriate attributes by analyzing attribute relations would indeed be an advancement, yet, manual validation by users remains essential. This ensures that no invalid PCEs are generated, which could negatively affect subsequent processes.

It should also be noted that the output of error messages is always dependent on the input PCE. If very complex PCEs with numerous errors are created, it may not be possible to correct them immediately. In such cases, the user must refine the PCE and validate it again, repeating the process until all errors are resolved iteratively. Users should be encouraged to construct manageable PCEs rather than attempting to encapsulate an entire medical history within a single PCE.

### Conclusions

In conclusion, this work demonstrates that the development of a comprehensive validation tool for postcoordinated SNOMED CT expressions can significantly enhance data quality and interoperability in health care, while also addressing and resolving versioning issues. The successful integration of the tool into existing systems highlights that it provides both syntactic and semantic support at a high level.

The creation of postcoordinated SNOMED CT expressions (PCEs) is not straightforward, as it requires adherence to the complex rules of the Compositional Grammar and the Concept Model. As seen in validations with real-world PCEs, ensuring syntactic and semantic accuracy can be challenging. The development of a validation tool for postcoordinated SNOMED CT expressions represents a significant advancement in addressing these challenges. In addition to technical validation, the tool provides helpful correction guidance, enabling the iterative improvement of PCEs. Users receive error reports and correction suggestions based on nine identified error categories. While the level of detail in the error messages can still be further optimized, the tool already offers valuable support for targeted and effective error resolution. The tool leverages state-of-the-art technologies such as the FHIR $validate-code service, the terminology server Ontoserver, and the SNOMED CT Concept Model. Ontoserver is indeed commercial, which poses a minor limitation. However, it was chosen because it is currently the only terminology server that supports postcoordination—a crucial criterion for the requirements of this project. The methodological approach combines intelligent error detection with tailored correction suggestions, making it easier for users to independently optimize their expressions. In this way, the tool makes a significant contribution to improving data quality in health care.

## Supplementary material

10.2196/67984 Multimedia Appendix 1 Usability survey.

## Abbreviations

ECL: Expression Constraint Language
FHIR: Fast Healthcare Interoperability Resource
MRCM: machine-readable Concept Model
PCE: postcoordinated expression
SNOMED CT: SNOMED clinical terms
