# Supplementary material for: A Validation Tool (VaPCE) for Postcoordinated SNOMED CT Expressions: Development and Usability Study
Source: JMIR Med Inform. 2025 Feb 28;13:e67984. doi: 10.2196/67984 (PMC11887581; doi:10.2196/67984)
Supplement: Multimedia Appendix 1 [file medinform-v13-e67984-s001.docx]

15188001 |Hearing loss (disorder)|:

{47429007 |Associated with (attribute)| = 285117005 |Noisy environment (environment)|}

The following SNOMED CT concept isn't included in the SNOMED edition and version being used: 285117005.

> What does this error mean?

One of the precoordinated concepts is inactivated in the SNOMED CT version and edition used (http://snomed.info/sct/900000000000207008/version/20240501).

> Can the inactivated concepts be replaced?

Replacement possible to create a syntactically and semantically correct PCE.

285117005->1186693005 |Noisy environment|

The following SNOMED CT concept isn't included in the SNOMED edition and version being used: 28139000.

> What does this error mean?

One precoordinated concept of the PCE doesn't exist in the selected version of SNOMED CT (http://snomed.info/sct/900000000000207008/version/20240501), because of an incorrectly entered Identifier.

> Can the incorrect concepts be replaced?

Replacement possible to create a syntactically and semantically correct PCE:

28139000->128139000| Inflammatory disorder (disorder)|

28139000 |Inflammatory disorder (disorder)|:

263502005 |Clinical course (attribute)| = 255227004 |Recurrent (qualifier value)|

Data basis (PCE and corresponding error message)

**User survey – Validation tool for postcoordinated SNOMED CT expressions**

Not MRCM compatible: 363704007 |Procedure site (attribute)| IS NOT a valid attribute for focus concept 284786005 |Able to wash self (finding)|.

> What does this error mean?

Each PCE or pre-coordinated concept can be assigned to a ConceptModel domain. These are usually sub-hierarchies of SNOMED CT. To guarantee medically meaningful expressions, SNOMED CT attributes are defined for a domain, which can be used to create SNOMED CT expressions. The SNOMED CT attribute 363704007 |Procedure site (attribute)| was used in the PCE shown, which is not allowed in the determined domain [Clinical finding (finding)].

> Which attribute does the attribute value match of the focus concept domain?

- Attribute: 363698007 |Finding site|

- Definition: |Finding site| specifies the body site affected by a condition.

- Range: << 442083009 |Anatomical or acquired body structure (body structure)|

- In domain of focus concept: true

- Suggestion: Replace the attribute 363704007 |Procedure site (attribute)| used with attribute
 363698007 |Finding site| to ensure a syntactically and semantically correct PCE.

284786005 |Able to wash self (finding)|:

{363704007 |Procedure site (attribute)| = 89545001 |Face structure (body structure)|}

Not MRCM compatible: 371154000 |Dependent (qualifier value)| is not in range for attribute 363703001 |Has intent (attribute)|

> What does this error mean?

A value range is a subset of SNOMED CT concepts that are valid for an attribute. The attribute value must be selected from this value range. This guarantees only meaningful attribute relations in terms of content or medicine. In the PCE shown, the attribute value 371154000 |Dependent (qualifier value)| is not contained in the value range of attribute 363703001 |Has intent (attribute)|.

> Which value range has the SNOMED CT attribute?

- Attribute: 363703001 |Has intent (attribute)|

- Definition: |Has intent| specifies the intent of a procedure.

- Range: << 363675004 |Intents (nature of procedure values) (qualifier value)|

> Which SNOMED CT attribute matches the selected attribute value?

Selected attribute value 371154000 |Dependent (qualifier value)| does not match with any value range of an attribute of the ConceptModel domain of the focus concept [Procedure (procedure)]. Change the attribute value or create a new PCE that can contain the selected attribute value.

- Attribute: 860779006 |Has ingredient characteristic|

- Definition: |Has ingredient characteristic| specifies a qualifer value.

- Range: << 362981000 |Qualifier value (qualifier value)|

- In domain of focus concept: false

---------

- Attribute: 363713009 |Has interpretation|

- Definition: |Has interpretation| when grouped with the attribute |interprets|, designates the judgment aspect being evaluated or interpreted for a concept.

- Range: << 260245000 |Finding value (qualifier value)| OR << 263714004 |Colors| OR << 308916002 |Environment or geographical location|

- In domain of focus concept: false

---------

- Attribute: 860781008 |Has product characteristic|

- Definition: |Has product characteristic| specifies a qualifier value.

- Range: << 362981000 |Qualifier value (qualifier value)|

- In domain of focus concept: false

737944006 |Care of urinary catheter (regime/therapy)|:

{363703001 |Has intent (attribute)| = 371154000 |Dependent (qualifier value)|}

Results

| **Question** | **Answer option** | **P1** | **P2** | **P3** | **P4** | **P5** |
| --- | --- | --- | --- | --- | --- | --- |
| How much do you know about SNOMED CT? | Nothing – Very much | 4 | 4 | 3 | 3 | 3 |
| How much do you know about SNOMED CT Postcoordination? | Nothing – Very much | 3 | 3 | 2 | 2 | 3 |
| How comprehensible is the error message in terms of language? | Very incomprehensible – Very comprehensible | 4 | 3 | 4 | 3 | 4 |
| How comprehensible is the error message in terms of structure? | Very incomprehensible – Very comprehensible | 3 | 3 | 4 | 3 | 4 |
| The level of detail for the error messages is appropriate. | Strongly disagree – Strongly agree | 2 | 3 | 4 | 2 | 4 |
| If not, why not? | – | …^1^ | …^3^ | – | – | – |
| How useful were the suggested corrections to fix the error? | Not useful at all – Very useful | 4 | 4 | 3 | 3 | 3 |
| How much do you trust the error diagnoses generated by the tool? | Not at all – Very strong | 3 | 4 | 4 | 4 | 4 |
| With the current error files as output: Would you use the tool regularly to validate PCEs? | Absolutely not – Absolutely | 3 | 3 | 4 | 4 | 5 |
| How useful would it be if the tool automatically created a corrected PCE (if possible)? | Not useful at all – Very useful | 5 | 5 | 5 | 5 | 5 |
| Further feedback | – | …^2^ | …^4^ | – | – | – |

^1^ “Too much information”

^2^ “As a user, it would be very much helpful if codes that run inactive in Snomed CT could be detected and corrected systematically.”

^3^ “Too much information, especially in the last example ‘Care of urinary catheter’”

^4^ “A correct output of the PCE would be very useful and helpful. A brief and understandable summary explaining why the provided code does not work would be sufficient. Especially in example 5, it only states that the code used is incorrect, but there is no indication of what needs to be done. In cases of incorrect codes, it has been helpful when the correct code is provided, as seen in example 1 'Inflammatory disorder' and 2 'Hearing loss'.”
